# Supplementary material for: Influence of Zooplankton and Environmental Factors on Clear-Water Phase in Lake Paldang, South Korea
Source: Int J Environ Res Public Health. 2021 Jul 5;18(13):7205. doi: 10.3390/ijerph18137205 (PMC8297077; doi:10.3390/ijerph18137205)
Supplement: Supplementary file 1 [file ijerph-18-07205-s001.zip › ijerph-1243006-supp.pdf]

Supplementary Materials

Influence of Zooplankton and Environmental Factors on Clear Water Phase in Lake Paldang, South Korea

Table S1. Correlation matrix showing coefficients between factors affecting the clear water phase in Lake Paldang.

Variables	TO-Ind	RO-Ind	CL-Ind	CO-Ind	RO-RA	CL-RA	CO-RA	Chl-*a*	SD	TUR	Temp	TN	TP	HRT	PRECI
TO-Ind	1														
RO-Ind	0.916**	1													
CL-Ind	0.407**	0.142**	1												
CO-Ind	0.504**	0.201**	0.452**	1											
RO-RA	0.154**	0.367**	-0.392**	-0.402**	1										
CL-RA	-0.036	-0.220**	0.594**	0.179**	-0.670**	1									
CO-RA	-0.156**	-0.307**	0.100*	0.422**	-0.770**	0.102*	1								
Chl-*a*	0.186**	0.167**	0.011	0.203**	0.038	-0.124*	0.076	1							
SD	-0.112*	-0.162**	0.154**	-0.037	-0.202**	0.330**	0.008	-0.468**	1						
TUR	-0.034	-0.063	-0.039	0.151**	-0.154**	0.070	0.106*	0.208**	-0.497**	1					
Temp	0.154**	0.088	0.289**	0.185**	-0.144**	0.250**	0.000	0.207**	0.050	0.137**	1				
TN	0.015	0.071	-0.221**	-0.116*	0.244**	-0.311**	-0.090	0.111*	-0.438**	0.161**	-0.534**	1			
TP	0.008	-0.002	0.004	0.043	-0.014	-0.042	0.053	0.389**	-0.567**	0.764**	0.161**	0.343**	1		
HRT	-0.015	-0.052	0.089	0.121*	-0.189**	0.112*	0.177**	-0.061	0.330**	-0.231**	-0.182**	-0.403**	-0.331**	1	
PRECI	-0.082	-0.070	0.016	-0.070	-0.024	0.081	-0.041	0.058	-0.377**	0.684**	0.199**	0.118*	0.662**	-0.371**	1

* $p < 0.05$, ** $p < 0.01$. TO: total; RO: Rotifera; CL: Cladocera; CO: Copepoda; Chl-*a*: chlorophyll-*a*; SD: secchi depth; TUR: turbidity; Temp: water temperature; TN: total nitrogen; TP: total phosphorus; HRT: hydraulic retention time; Ind: individuals; RA: relative abundance; PRECI: precipitation.
